# Long-term exposure to ambient air pollution and measures of central hemodynamics and arterial stiffness among multiethnic Chicago residents

**DOI:** 10.1186/s12940-024-01077-z

**Published:** 2024-05-07

**Authors:** Saira Tasmin, Briseis Aschebrook-Kilfoy, Donald Hedeker, Rajan Gopalakrishnan, Elizabeth Connellan, Muhammad G. Kibriya, Michael T. Young, Joel D. Kaufman, Habibul Ahsan

**Affiliations:** 1 Department of Public Health Sciences, University of Chicago, 5815 S. Maryland Ave, Rm TC-620A, MC2000, Chicago, IL 60637 USA; 2 Institute for Population and Precision Health, University of Chicago, Chicago, IL USA; 3 Comprehensive Cancer Center, University of Chicago, Chicago, IL USA; 4 Department of Environmental & Occupational Health Sciences, University of Washington, Seattle, WA USA

## Abstract

**Objectives:**

To examine whether long-term air pollution exposure is associated with central hemodynamic and brachial artery stiffness parameters.

**Methods:**

We assessed central hemodynamic parameters including central blood pressure, cardiac parameters, systemic vascular compliance and resistance, and brachial artery stiffness measures [including brachial artery distensibility (BAD), compliance (BAC), and resistance (BAR)] using waveform analysis of the arterial pressure signals obtained from a standard cuff sphygmomanometer (DynaPulse2000A, San Diego, CA). The long-term exposures to particles with an aerodynamic diameter < 2.5 μm (PM2.5) and nitrogen dioxide (NO2) for the 3-year periods prior to enrollment were estimated at residential addresses using fine-scale intra-urban spatiotemporal models. Linear mixed models adjusted for potential confounders were used to examine associations between air pollution exposures and health outcomes.

**Results:**

The cross-sectional study included 2,387 Chicago residents (76% African Americans) enrolled in the ChicagO Multiethnic Prevention And Surveillance Study (COMPASS) during 2013–2018 with validated address information, PM2.5 or NO2, key covariates, and hemodynamics measurements. We observed long-term concentrations of PM2.5 and NO2 to be positively associated with central systolic, pulse pressure and BAR, and negatively associated with BAD, and BAC after adjusting for relevant covariates. A 1-µg/m^3^ increment in preceding 3-year exposures to PM2.5 was associated with 1.8 mmHg higher central systolic (95% CI: 0.98, 4.16), 1.0 mmHg higher central pulse pressure (95% CI: 0.42, 2.87), a 0.56%mmHg lower BAD (95% CI: -0.81, -0.30), and a 0.009 mL/mmHg lower BAC (95% CI: -0.01, -0.01).

**Conclusion:**

This population-based study provides evidence that long-term exposures to PM2.5 and NO2 is related to central BP and arterial stiffness parameters, especially among African Americans.

**Supplementary Information:**

The online version contains supplementary material available at 10.1186/s12940-024-01077-z.

## Introduction

There is a well-established relationship between ambient air pollution (AAP) exposure and cardiovascular disease (CVD) morbidity and mortality; however, the underlying mechanisms are poorly understood [1, 2]. One potential mechanism is an effect of air pollution on blood pressure (BP), mediated through autonomic nervous system alterations and/or changes in inflammation and oxidative stress [3]. Some recent epidemiological studies demonstrated an increase in peripheral BP associated with long-term exposure to particulate matter (PM2.5) [4, 5] and black carbon exposure [6], however, the associations of AAP with central BP has less investigated. Central BP markers are independent risk factors for CVD and may better predict cardiovascular events and mortality than brachial BP. Central hemodynamics can now be reliably assessed non-invasively with a number of relatively inexpensive devices. One study evaluated the associations between personal exposure to air pollution from biomass stoves and central hemodynamic parameters in 205 rural Chinese women and found that personal exposures to air pollution were associated with higher brachial and central BP and lower pulse pressure amplification in older Chinese women using biomass stoves [7]. Another panel study of 65 nonsmoking patients with metabolic syndrome in China evaluated the relationship of AAP with central hemodynamics and demonstrated that short- to medium-term exposures to high levels of ambient air pollution adversely impacted central hemodynamics [8].

Increased arterial stiffness, another measure of vascular dysfunction, is a risk factor for the development of cardiovascular events [9]. One method to examine vascular stiffness non-invasively is based on brachial artery distensibility (BAD), compliance (BAC) and resistance (BAR) measures [10]. A lower BAD and BAC (and higher BAR) indicate increased arterial stiffness, which have been suggested as reflecting the arteriosclerosis burden [11]. Lower BAD and BAC are also associated with an elevated level of coronary artery calcium (CAC) [11, 12], a marker of subclinical artery atherosclerosis. To date, studies of long-term air pollution exposure and arterial stiffness have reported inconsistent results [13–15] and results differed according to which measure of arterial stiffness was examined [14]. Differences in study populations, location and exposure measurements also may contribute to diverse findings on associations between PM and arterial stiffness across studies [16]. Most studies used measures such as augmentation index and carotid femoral pulse wave velocity; however, very little is known about potential role of long-term exposure to air pollution on altered brachial artery stiffness measures using waveform analysis of the arterial pressure signals.

In this study, we aim to evaluate the exposure–response associations of long-term exposure to AAP with measures of central hemodynamics and brachial artery stiffness using pulse waveform analysis in a recently established multiethnic population-based U.S. cohort in Chicago.

## Methods

The data that support the findings of this study are available from the corresponding author upon reasonable request.

### Study population

This study includes participants who participated in the Chicago Multiethnic Prevention And Surveillance Study (COMPASS). COMPASS is a population-based longitudinal cohort study with ongoing recruitment investigating how lifestyle, environmental, biological and other factors impact the risk of chronic diseases. Recruitment methods of COMPASS cohort have been described elsewhere [17, 18]. The current study included 2,387 participants 35–94 years of age who were enrolled into COMPASS during 2013–2018 and underwent in-home hemodynamics measurements by trained research staff and have valid address information, PM2.5 or NO2 estimates, key covariates, and hemodynamics measurement. The COMPASS study was approved by the Institutional Review Board (IRB) of the University of Chicago; all participants provided informed consent.

Face to face interviews were administered by trained staff who collected information on participant demographics, socioeconomic status, residential history, occupational history, personal medical history (including self-reported diabetes, heart attack, sleep apnea and hypertension), medication use, perceived stress, and lifestyle factors such as alcohol use and smoking. Participants were asked whether they had ever been diagnosed with certain diseases by a physician, including diabetes mellitus and hypertension. Responses were self-reported as no, yes, or refused/don’t know. Hypertension was defined as either self-reported physician-diagnosed hypertension or self-reported current intake of any antihypertensive medication, or both.

### Hemodynamic measurement

Participant’s hemodynamic parameters were measured in their homes and according to guidelines using DynaPulse 2000 A, a noninvasive central BP and hemodynamic monitoring instrument (Pulse Metric, Inc., San Diego, CA). Before measurements were collected, study participants were seated for 5 min, and then the device’s cuff was placed around their upper arm. The correct cuff size for the patient was determined from small, medium, and large in accordance to American Heart Association (AHA) guideline recommendation that the rubber bladder inside the cuff should encircle 80% of the arm in adults [19]. This yielded an array of central hemodynamic variables including:

1. Central (aortic) BP parameters [central systolic and diastolic pressures (cSBP, cDBP), pulse pressure (cPP), and mean arterial pressure (cMAP)]: blood pressure measures central arterial blood pressure at end systole and diastole. Measured using proprietary Pulse Dynamics waveform pattern recognition algorithms.
2. Cardiac parameters: cardiac output (CO), left ventricular ejection time and contractility, and stroke volume.
3. Brachial artery stiffness parameters: Brachial artery compliance, distensibility and resistance (BAC, BAD, BAR).
4. Systemic vascular compliance and resistance (SVC, SVR).

The DynaPulse 2000 A derives brachial artery measures using waveform analysis of the arterial pressure signals obtained from a cuff sphygmomanometer [20]. In brief, measurements from the oscillometric signal are incorporated into a physical model of the brachial artery. The model assumes a straight tube brachial artery and T tube aorta, and assumes that the systolic phase of the suprasystolic wave and diastolic phase of the subdiastolic wave closely approximate systolic and diastolic aortic pressures, respectively. DynaPulse’s pattern-recognition algorithm identifies the signal changes in phase that correspond to systolic, diastolic and mean arterial pressures based on the dynamic effect of blood flow past the cuff [21–23]. DynaPulse also has been previously validated with high correlation between compliance measurements obtained during cardiac catheterization at University of California San Diego Medical Center Cath-Lab and showed good agreement for noninvasive cardiovascular hemodynamics comparing to catheterization data (*r* = 0.83) [21, 24]. DynaPulse also has been previously validated with high correlation between compliance measurements obtained during cardiac catheterization and noninvasive brachial methods (*r* = 0.83) [21, 22]. Reproducibility studies using blind duplicates demonstrated good intraclass correlation coefficients for arterial compliance, from which distensibility is calculated (0.72) [23]. Two recordings were performed sequentially, and measurements were obtained for systolic, diastolic, and mean arterial BP as well as heart rate. All measurements were averaged. Off-line analyzes of brachial artery pressure curves are performed using an automated system.

### Measurement of exposure to air pollutants

Annual average concentrations of air pollutants for each participant’s address were estimated by spatiotemporal models developed for Chicago communities by the Multi-Ethnic Study of Atherosclerosis (MESA) Air Pollution study (MESA Air), which have been described previously [24]. MESA Air predicted ambient (household-level) concentrations of particulate matter less than 2.5 μm in diameter (PM2.5) and oxides of nitrogen (NO2) using residence-specific hierarchical spatiotemporal models in Chicago and elsewhere [25]. Briefly, this modeling approach used daily air pollution concentrations collected from the regulatory networks of U.S. Environmental Protection Agency (EPA) Air Quality System (AQS) supplemented with observed concentrations of pollutants obtained from an intensive monitoring campaign of samples collected in the communities and at the homes of the MESA cohort. Moreover, these models had taken into account the relevant geographic covariates such as land use, local emission sources, and population density at or near participants’ home addresses and the correlation of concentrations across space to estimate concentrations to provide fine-scale spatial predictions of PM2.5 and NO2.

Each participant’s residential address was geocoded into latitude and longitude data. Based on the residential geocodes, census blocks were assigned to each participant. Individual long-term exposure to ambient PM2.5 and NO2 pollution was assigned from the census block specific mean annual average PM2.5 and NO2 levels (for the 3 calendar years prior to each participant’s enrollment) estimated by MESA Air .

### Statistical analysis

Participants’ addresses were geocoded to obtain geographic information system (GIS) information using ArcGIS (California, ESRI). To adjust for potential clustering of the outcome on a small-scale spatial level, linear mixed models were used to examine associations between AAP exposures and various measures of hemodynamic variables where we included a random intercept for the U.S. census block. These models account for any clustered data and control for socioeconomic status at the neighborhood level (here, by census block). For our analytic data sets, we restricted our study population to only those participants with valid address information, PM2.5 or NO2 estimates, key covariates, and hemodynamics measurement.

The *a priori* selection of covariates was based on a review of the literature before the analysis to avoid model selection bias. We initiated the model development with a crude model (no variable adjustment), and then added a range of covariates into successive regression models. The final models included age, sex and race [African American, or others (others include White, Indian (American), Alaskan Native, Native Hawaiian, Guamanian, Samoan, Other Pacific Islander, Asian Indian, Chinese, Filipino, Japanese, Korean)], individual level socioeconomic status: (income, education, marital status), CVD risk factors [body mass index (BMI), waist, smoking status (current smoker, former or never smoker), alcohol consumption (None or < 1 drink/week, 1–3 drinks/week, or > 3 drinks/week)], season, history of Type 2 diabetes (classified as self-reported physician-diagnosed diabetes) and history of hypertension (classified as self-reported physician-diagnosed hypertension or current intake of any antihypertensive medication, or both). The final model analyses were also restricted to individuals with stable residence (i.e., only participants who lived at their current address for more than 3 years) to account for potential exposure misclassification arising from characterizing current residence as a location of long-term exposure.

When we observed significant associations with AAP exposure in the final model, we additionally examined heterogeneity by BMI (normal, overweight and obese), race (African American and others), sex (Female and male), age (≤ 60 years and > 60 years) and smoking status (current smoker and former or never smoker) by stratified analysis of air pollution and outcome hemodynamic variables and assessed statistical significance for heterogeneity [26].

Natural cubic splines were used for PM2.5 and NO2 to check whether the exposure-response relationships were linear or nonlinear. Log-transformed terms of air pollutants were used to check if log-transformed terms were a better fit. Separate models were conducted for PM2.5 or NO2 due to their high correlation (*r* = 0.84). Because there may be spatially varying characteristics that we were unable to account for, sensitivity analyses included varying the number of degrees of freedom (df) for spatial adjustment and investigating the impact on main effect sizes and standard errors of alternate forms of the other independent and dependent variables. We also conducted sensitivity analyses excluding participants taking anti-hypertensive medicines to examine impact on the associations. All analyses were completed using STATA (StataCorp LP, College Station, TX).

## Results

### Participant characteristics

Table 1 presents baseline demographic characteristics and Table 2 shows baseline hemodynamic characteristics of participants. Among the 2,387 participants, the mean ± SD age was 53 ± 11 years and a nearly equal sex distribution. Of all study subjects, the mean BMI was 29 ± 8 kg/m^2^. 44% of participants reported a history of hypertension and 36% of participants were on anti-hypertensive medications. Mean ± SD cSBP was143 ± 20 (25 percentile 129; 75 percentile 155) and cDBP was 82 ± 13 mmHg (25 percentile 72; 75 percentile 89). Average BAD, BAC and SVC were 0.08 mL/mmHg, 5.74%mmHg and 1.29 mL/mmHg, respectively.

**Table 1:** Demographic characteristics of the study population (*n* = 2,387)

Variable	Mean	SD
Age (yrs)	53	11
Waist (inch)	38	6
BMI (kg/m^2^)	29	8
Residence years	3.6	2
	**Number**	**%**
Sex		
Male	1,136	48
Female	1,251	52
Race		
Others	576	24
African Americans	1,804	76
Education		
High school or less	1,286	54
Some college	833	35
Bachelors or above	264	11
Income		
Less than $20,000	1,377	64
$20,000-$49,999	525	24
$50,000 or above	265	12
Smoking status		
Current smoker	1,220	51
Former smoker	427	18
Never smoker	738	31
Alcohol consumption (/per week)		
> 3 drinks/week	1,254	60
1–3 drinks/week	247	12
None or less than once/week	600	29
Prevalent hypertension		
Yes	1,057	44
No	1,326	56
Presence of Type2 diabetes		
Yes	307	13
No	2,075	87

**Table 2:** Hemodynamic characteristics of the study population (*n* ~ 2,387)

Variables		Mean		Std. Dev.	Min	Max	
Central BP parameters							
Systolic blood Pressure (cSBP)(mmHg)	143		20		84	200	
Diastolic blood Pressure (cDBP) (mmHg)	82		13		50	118	
Pulse pressure (cPP) (mmHg)	61		14		25	99	
Mean arterial blood pressures (cMAP) (mmHg)	102		15		61	174	
**Cardiac parameters**							
Left ventricular ejection time (sec)	0.26		0.04		0.14	0.44	
Left ventricular Contractility (1/s)	14.84		1.77		9.16	22.07	
Cardiac output (L/min)	5.97		1.79		2.91	18.00	
Cardiac Index (L/min/m2)	3.09		0.73		1.50	6.98	
Stroke Volume (mL)	76		17		36	196	
Stroke Volume Index (mL/m2)	39		6		25	72	
**Systemic vascular parameters**							
Systemic Vascular Compliance (mL/mmHg)	1.29		0.32		0.50	3.36	
Systemic Vascular resistance (kdnes/sec/cm5)	1,458		403		394	3,171	
**Brachial artery parameters**							
Brachial artery compliance (BAC) (mL/mmHg)	0.08		0.03		0.02	0.26	
Brachial artery distensibility (BAD) (%mmHg)	5.74		1.44		1.92	13.97	
Brachial artery resistance (BAR) (kdnes/sec/cm5)	185		99		25	884	

### Residential pollutant exposures

Table 3 shows the 3-year average concentrations of air pollutants for the 2,387 participants. The estimated 3-year average concentrations(previous 3 calendar years of each participant’s enrollment) of PM2.5 and NO2 at participant addresses were 11.2 µg/m^3^ and 16 ppb, respectively, which were below the National Ambient Air Quality Standards of the US Environmental Protection Agency (12 µg/m^3^ for PM2.5 and 53 ppb for NO2).

**Table 3:** Distribution of participants 3-y average residential PM2.5 and NO2 concentrations

Exposures	Mean	SD	Min	Max
PM2.5 (µg/m^3^)	11.18	0.42	9.88	12.02
NO2 (ppb)	16.03	2.02	11.16	19.62

### Relationship of pollutants, central BP and cardiac parameters

We evaluated the association of central BP and cardiac parameters with long-term air pollution concentrations in crude models and after adjusting for potential covariates, and expressed the results as the estimated change associated with one unit increase in concentration (1 µg/m^3^ for PM2.5, and 1 ppb for NO2 here). Table 4 shows the results of adjusted mixed effect models by each pollutant. In the adjusted models, preceding 3-year exposures to PM2.5 were significantly associated with cSBP, cPP and cMAP, but not with cDBP (Table 4). The effect estimates of cSBP were 1.875 (95% CI:0.985, 4.156) and cPP were 1.014 (95% CI: 0.419, 2.865) mmHg with increments of 1 µg/m^3^ for PM2.5. The cardiac parameters were not significantly associated with PM2.5 and NO2. In crude models, both PM2.5 and NO2 were positively associated with cPP, and only PM2.5 was positively associated with cSBP and cMAP (Supplemental Table 1).

**Table 4:** Estimated change in Hemodynamic variables with 95% CI per unit change of long-term exposure (preceding 3 year averages) to the air pollutants (PM2.5 and NO2)

	PM_2.5_				NO_2_			
	Estimate	95%CI		p-value	Estimate	95%CI		p-value
**Central BP parameters**								
cSBP	1.875	0.985	4.156	0.018	0.676	-0.045	1.397	0.066
cDBP	1.181	-0.650	3.013	0.206	0.094	-0.255	0.443	0.596
cPP	1.014	0.419	2.865	< 0.001	0.729	0.258	1.201	0.002
cMAP	1.479	0.433	3.862	0.006	0.338	-0.063	0.738	0.099
**Cardiac parameters**								
Left ventricular ejection time	0.002	-0.006	0.010	0.647	0.001	-0.001	0.003	0.215
Left ventricular contractility	0.092	-0.274	0.458	0.622	0.032	-0.042	0.105	0.399
Cardiac output	0.076	-0.095	0.247	0.383	0.001	-0.034	0.037	0.941
Cardiac Index	0.046	-0.041	0.134	0.301	0.007	-0.011	0.024	0.444
Stroke Volume	-0.061	-1.590	1.467	0.937	0.003	-0.305	0.311	0.983
Stroke Volume Index	0.443	-0.158	1.043	0.148	0.100	-0.014	0.214	0.085
**Brachial artery parameters**								
BAD	-0.557	-0.809	-0.304	< 0.001	-0.089	-0.145	-0.034	0.002
BAR	23.697	9.687	37.707	0.001	3.590	0.616	6.564	0.018
BAC	-0.009	-0.013	-0.005	< 0.001	-0.001	-0.002	-0.001	0.001
**Systemic vascular parameters**								
SVC	-0.094	-0.140	-0.048	< 0.001	-0.012	-0.022	-0.001	0.026
SVR	35.070	-20.624	90.764	0.217	4.019	-7.560	15.597	0.496

*Final models with participants having stable residence history and adjusted for age, BMI, waist, race, sex, smoking status, alcohol consumption, education, income, marital status, season, Type 2 diabetes, and hypertension.

### Relationship of air pollutants, brachial artery parameters and systemic vascular parameters

The preceding 3-year concentrations of both PM2.5 and NO_2_ were associated with lower BAD, BAC and SVC and higher BAR. A 1-µg/m3 increment in PM2.5 was associated with a 0.557%mmHg lower BAD (95% CI: -0.809, -0.304), and a 0.009 mL/mmHg lower BAC (95% CI: -0.013, -0.005). A 1-ppb increase in NO_2_ was associated with a 0.089%mmHg lower BAD (95% CI: -0.145, -0.034), and a 0.001 mL/mmHg lower BAC (95% CI: -0.002, -0.001) (Table 4). The associations between SVC and a unit increment of PM2.5 and NO2 were − 0.094 ml/mmHg (95% CI: − 0.140, -0.048) and − 0.012 ml/mmHg (95% CI: − 0.022, -0.001), respectively. PM2.5 and NO2 were not significantly associated with SVR. Similar magnitude and trend were also found for BAD, BAC and SVC in crude models, only the association of BAR and PM was not significant in the crude models (Supplement Table 1).

### Effect modification

**Fig. 1:**
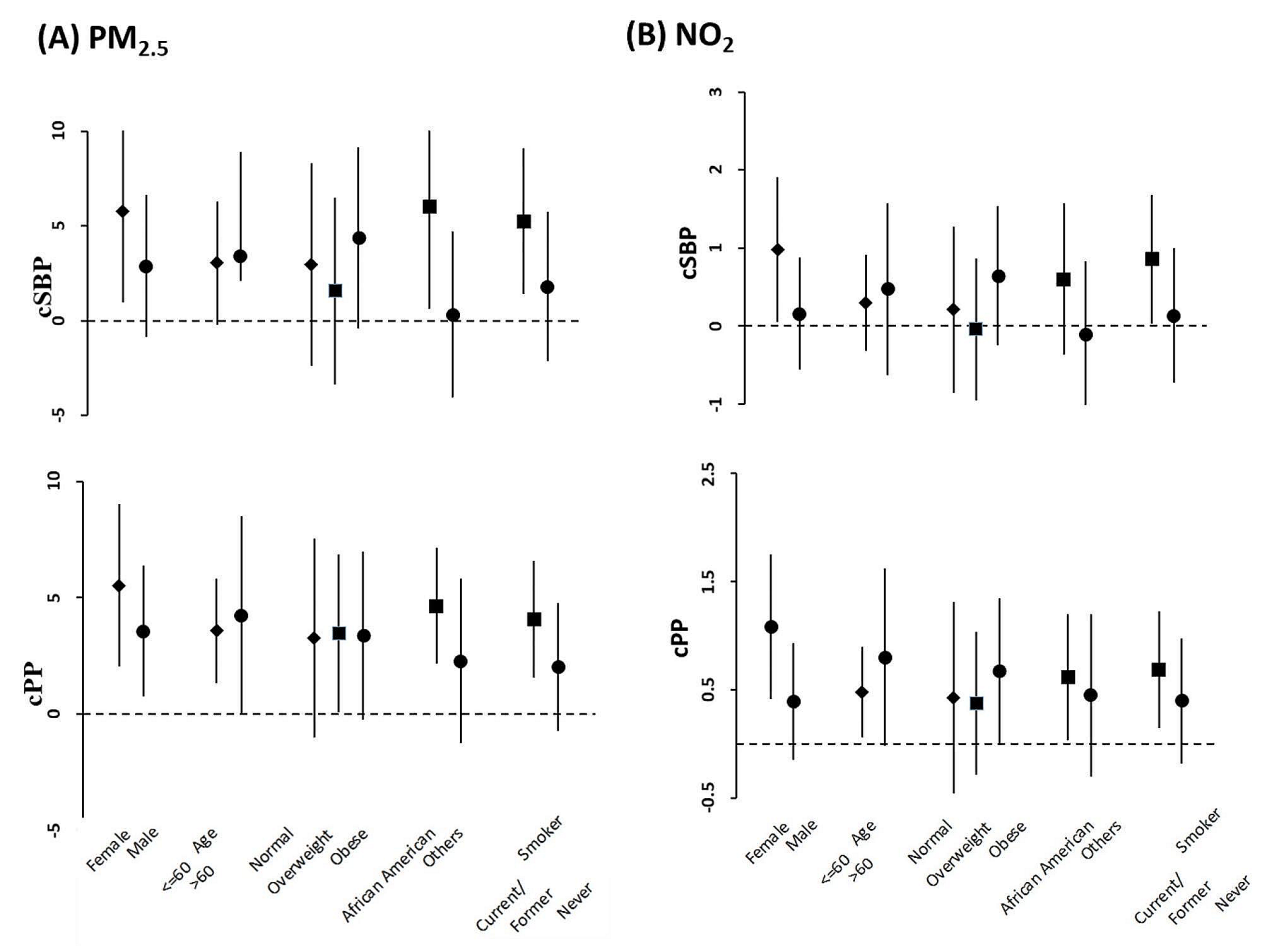
Stratified analysis on associations between long-term PM2.5 (**A**) and NO2 (**B**) exposure (unit increments, 1 µg/m^3^ for PM2.5, and 1 ppb for NO2) and systolic blood pressure (cSBP), and pulse pressure (cPP). Effect estimates (coefficients) are derived from mixed effect analysis, and bars cover 95% confidence intervals. Results were obtained from final models with participants having stable residence history and adjusted for age (not in age-stratified analysis), BMI (not in BMI-stratified analysis), race (not in race-stratified analysis), sex (not in sex-stratified analysis), smoking status (not in smoking status-stratified analysis), waist, alcohol consumption, education, income, marital status, season, Type 2 diabetes, and hypertension

**Fig. 2:**
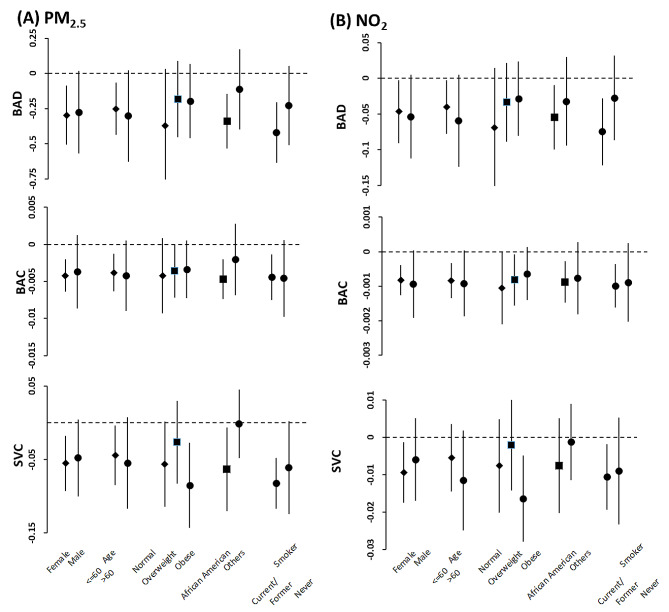
Stratified analysis on associations between long-term PM2.5 (**A**) and NO2 (**B**) exposure (unit increments, 1 µg/m^3^ for PM2.5, and 1 ppb for NO2) and brachial artery distensibility (BAD), brachial artery compliance (BAC) and systemic vascular compliance (SVC). Effect estimates (coefficients) are derived from mixed effect analysis, and bars cover 95% confidence intervals. Results were obtained from final models with participants having stable residence history and adjusted for age (not in age-stratified analysis), BMI (not in BMI-stratified analysis), race (not in race-stratified analysis), sex (not in sex-stratified analysis), smoking status (not in smoking status-stratified analysis), waist, alcohol consumption, education, income, marital status, season, Type 2 diabetes, and hypertension

We examined heterogeneity by BMI, race, sex, age and smoking status in fully adjusted models by stratified analyses for the outcomes, which had significant associations with air pollutants (Figs. 1 and 2). There was no significant evidence of effect modification by sex (Female vs. male), BMI (Normal vs. overweight, or normal vs. obese), or age ( < = 60 vs. > 60). However, stratified analyses by race revealed that African American participants had a higher estimated risk of having higher SBP and lower BAD, or SVC for their associations with PM2.5 compared to other race participants (Supplemental Tables 2 and 3). In addition, stratifying the data by smoking status (current/former vs. never) showed that current or former smokers had excess estimated risk of having higher SBP and lower BAD or SVC for their associations with air pollution compared to never smokers, although not statistically significant.

### Sensitivity analyses

Varying the df for spatial adjustment had little impact on the associations of hemodynamic parameters with PM2.5 and NO2. Using natural logarithmic transformations of the exposure and outcome variables produced no appreciable changes in the overall findings of the analysis (data not shown). The results of sensitivity analyses using splines to assess nonlinearity of associations between hemodynamic measures and the exposures of interest were generally consistent with linearity, with little evidence of nonlinearity (data not shown). The sensitivity analyses showed that exclusion of the participants taking anti-hypertensive medicine did not change the results substantially (Supplemental Table 4).

## Discussion

In this large-scale population-based study, we found evidence that long-term exposure to AAP (PM2.5 and NO2) was associated with higher central cSBP and cPP, and lower BAD, BAC and SVC among multiethnic participants in Chicago. Results were robust across model selections and in sensitivity analyses. We observed a large effect size for PM2.5–central cSBP association in our study and the adverse effects were significantly stronger in African Americans than in other races.

Central BP is the pressure in the aorta, the artery that sends blood from the heart throughout the body. The clinical importance of standard brachial cuff measurement of peripheral BP is undeniable; however, peripheral BP may be an inaccurate substitute for central BP [27]. An independent prognostic value of central BP has been illustrated in larger studies using non-invasive central BP methods, while yet to be fully adopted in clinical practice [28–31]. Few studies have examined the relationship between long-term ambient exposure to both PM2.5 and NO2 and BP, and to the best of our knowledge, none has evaluated central BP.

Positive associations between long-term exposure to AAP and peripheral BP have been observed previously. Exposure to PM2.5 was reported to be associated with both peripheral systolic and diastolic blood pressure (pSBP and pDBP) [4, 32, 33]. Schwartz et al. (2012) observed an increase in pSBP and pDBP of 2.6 mm Hg (95% CI: 1.4; 3.8) and 2.4 mm Hg (95% CI: 1.8; 3.1) respectively, per IQR (0.3 µg/m^3^) increase in the previous year black carbon levels among 853 U.S. veterans [34]. In the Sister Study, conducted among 43,629 women of 35–76 years age, a 10-µg/m^3^ increase in PM2.5 was associated with 1.4-mmHg higher pSBP (95% CI: 0.6, 2.3; *p* < 0.001), 1.0-mmHg higher pPP (95% CI: 0.4, 1.7; *p* = 0.001), and a 10-ppb increase in NO2 was associated with a 0.4-mmHg (95% CI: 0.2, 0.6; *p* < 0.001) higher pPP [4, 35]. Long-term average PM2.5 was also shown to be associated with increased peripheral BP in a population-based cohort study (*n* = 4,291) in a single metropolitan area in western Germany [5]. In Taiwan, a study showed strong positive associations between peripheral BP and both annual average PM2.5 and NO2 [32]. In contrast, a Danish population-based cohort study (*n* = 57,053) found a small reduction in pSBP with long-term average NOx exposure [36]. A study of Chinese adults (*n* = 24,845) found no relationship between nearest monitor NO2 and peripheral BP, but did find small increases in l pSBP and pDBP in men associated with changes in PM10, SO2, and O3 [37]. In the MESA prospective cohort, positive associations were found between long- or short-term exposures to AAP and peripheral BP in older adults without baseline clinical cardiovascular disease only in models with adjustment for time-varying age but not after accounting for both time-varying age and calendar time [38]. We observed larger effect size for PM2.5-cSBP association in our study compared to the most previous studies reporting PM-peripheral pSBP associations. However, a direct comparison of the estimated magnitudes of our findings with those from other studies is limited by the difference in expression of BP measures i.e. central vs. peripheral BP. Moreover, similar to our study, large effect estimate was observed in a recent cross-sectional study where a 1 µg/m^3^ increase in ambient PM2.5 was found to be associated with 1.4 mm Hg higher pSBP (95%CI: 0.12, 2.7) in women in India [39]. To explain this, these authors suggested that the possible explanations for this variation could be the high prevalence of underlying hypertension, within-cluster difference in effect estimate of PM2.5 exposure on BP rather than between-cluster (e.g., between-city) exposure, and fine-scale air pollution exposure assessment.

We found that the African Americans had stronger association of central cSBP and PM2.5 compared to other races. There are well-established disparities in cardiovascular health outcomes between minority and non-minority groups across the U.S., especially in Chicago. African Americans have a greater burden of myocardial infarction, heart failure, stroke, and other cardiovascular events [40]. African Americans tend to have higher BP and exhibit lower levels of BP control than whites in the U.S., even after consideration of modifiable health behaviors, suggesting that other racial/ethnic differences underlie these disparities [41]. Moreover, African descent populations were also found to have increased central arterial stiffness throughout different life stages compared with the white European-origin people in UK [42]. Although few studies have evaluated the modifying role of race on the association between PM2.5 and peripheral BP, researchers have examined other CVD outcomes, with mixed results. For example, in a community-based cohort in Western Pennsylvania, African Americans had higher rate of CVD events compared with whites that was partly explained by higher exposure to PM2.5 [43]. On the contrary, using data from the National Health Interview Survey, Parker et al. (2018) showed that the associations between air pollution and heart disease mortality for non-Hispanic black and Hispanic adults were not statistically significantly different from that for non-Hispanic white adults after controlling for sociodemographic and geographic factors [44]. Our findings suggest that African Americans are more vulnerable to long-term air pollution effects and this finding of a higher risk among African Americans has important implications for policy makers to set standards that protect sensitive populations.

In addition to central BP, we also observed long-term exposure to PM2.5 and NO2 to be associated with brachial artery measures (negatively associated with BAD and BAC, and positively associated with BAR) in this study. As lower BAD and BAC (and higher BAR) indicate increased arterial stiffness, our results demonstrated adverse effects of PM2.5 and NO2 on arterial stiffness parameters. In line with our study, previous studies have also demonstrated adverse effects of higher long-term exposure to PM on various measures of arterial stiffness [45]. Arterial stiffness measured as distensibility, Young’s elastic modulus (YEM) and stiffness index was found to be associated to elevated prenatal exposures to PM2.5 and PM10 in young adults [45]. Long-term exposure to environmental air pollution also showed adverse vascular effects measured as brachial-ankle pulse wave velocity (PWV) in specific subpopulations such as physically inactive older adults [46], and patient undergoing hemodialysis [47]. However, in contrast to our study, the long-term estimate of PM2.5 exposure was not associated with the carotid-femoral pulse-wave velocity (CFPWV) measure of arterial stiffness among participants in the Framingham Heart Study [13]. The findings from the MESA, including about 4000 participants from 6 US cities also found no associations between estimated 20-year exposure to PM10 or PM2.5 and carotid stiffness, assessed by Young’s modulus measure of elasticity [15]. Similarly, long-term PM2.5 was not associated with radial artery derived PWV or augmentation index in a study of 745 young adults in a Dutch study [48].

A reproducible, noninvasive method for measuring arterial stiffness was used in this study based on brachial artery pulse waveform analysis algorithms. These new techniques, which utilize different aspects of the pulse pressure waveform, are simple, reliable, useful in measuring subclinical vascular changes related to arteriosclerosis, and may identify patients at increased risk of developing cardiovascular complications [49]. Vascular changes in brachial artery are independent of aging, since stiffness in the brachial artery shows less change with age compared to large elastic arteries [50]. As both hypertension and atherosclerosis are associated with structural and functional vascular changes, this method of compliance and distensibility measurement in relation to long-term air pollution exposure could be of clinical value and useful for identifying subjects at particular risk of cardiovascular complication.

Primary strengths of this study include its population-based recruitment approach with high participation of minority participants, large sample size, high-quality noninvasive measures central hemodynamic and brachial artery parameters, detailed characterization of potential confounders including individual and neighborhood-level SES and the use of modeled fine-scale intra-urban estimates of exposure to both PM2.5 and NO2. This study used state-of-the-art, individual-level exposure estimates derived from spatiotemporal models using extensive project-specific measurements [24, 25]. As with all modeled exposures, however, there is exposure measurement error in this study that may influence the magnitude or significance of our results. This is a limitation in any air pollution study using stationary monitors or modelled exposures because the true individual exposure is not measured.

A major limitation of this study is the cross-sectional design. Thus, no causal inference can be made. It is possible that high central BP or low brachial artery parameters preceded the AAP exposure. Dynapulse derived hemodynamic measurements are estimations by oscillometric cuff signal pattern recognition. Although arterial pressures and stiffness measurements obtained by Dynapulse were validated with standard and invasive measurements, and good agreement was observed between the methods [20, 21, 51]; Dynapulse measurements are estimated from mathematical transformation and thus errors cannot be excluded. Furthermore, Dynapulse measurements only measured one vascular bed, and the calculated resistance may vary in different vascular beds simultaneously [9]. Moreover, although we did control for SES at the individual level and at the neighborhood level by using mixed effect models to account for clustering by census tract, it is likely that the number of years of education does not provide strong enough control of individual-level SES; hence, there may still be some residual confounding by SES. In addition, our utilization of air pollution concentrations derived from spatiotemporal models may have led to exposure misclassification. This is because the estimates obtained from residential addresses might not accurately reflect individual exposure levels and do not account for time spent away from home. There is potential for confounding by other exposures such as indoor sources, as we only measured personal smoking and not whether there was another smoker in the home. Overall, we have included most key confounders in our model so we do not expect that unmeasured confounding would have a strong influence on our results.

## Conclusion

Evidence from this population-based study provides evidence that long-term exposure to ambient air pollution, specifically PM2.5 and NO2, is related to central BP and arterial stiffness parameters, especially among African Americans. Our results suggest that higher central BP, reduction in brachial arterial and systemic vascular compliance — may play a mediating role in associations previously observed between air pollution and cardiovascular disease. We believe that this study provides an important contribution to the evidence on the association between air pollution and cardiovascular morbidity and to the identification of subpopulations at highest risk.

### Electronic Supplementary Material

Below is the link to the electronic supplementary material.

Supplementary Material 1

## Data Availability

The data that support the findings are not publicly available due to ethical consideration (privacy of research participants). The data of this study are available from the corresponding author upon reasonable request.

## Abbreviations

AAP: ambient air pollution
BP: blood pressure
CVD: cardiovascular disease
PM: Particulate matter
BAD: brachial artery distensibility
BAC: brachial artery compliance
BAR: brachial artery resistance
CAC: coronary artery calcium
COMPASS: Chicago Multiethnic Prevention And Surveillance Study
IRB: Institutional Review Board
AHA: American Heart Association
cSBP: central systolic pressure
cDBP: central diastolic pressure
cPP: central pulse pressure
cMAP: central mean arterial pressure
CO: cardiac output
SVC: systemic vascular compliance
SVR: systemic vascular resistance
LVC: left ventricular contractility
MESA Air: Multi-Ethnic Study of Atherosclerosis Air Pollution study
PM2.5: Particulate matter with diameter less than equal to 2.5 μm
NO2: Nitrogen dioxide
EPA: Environmental Protection Agency
AQS: Air Quality System
GIS: geographic information system
BMI: body mass index
YEM: Young’s elastic modulus
PWV: pulse wave velocity
CFPWV: carotid-femoral pulse-wave velocity
