# Supplementary material for: Long-term exposure to ambient air pollution and measures of central hemodynamics and arterial stiffness among multiethnic Chicago residents
Source: Environ Health. 2024 May 7;23:47. doi: 10.1186/s12940-024-01077-z (PMC11075200; doi:10.1186/s12940-024-01077-z)
Supplement: Supplementary file 1 — Supplementary Material 1 [file 12940_2024_1077_MOESM1_ESM.docx]

**Supplementary Material:** **Long-term exposure to ambient air pollution and measures of central hemodynamics and arterial stiffness among multiethnic Chicago residents.**
